# The normal electrocardiograms in the conscious newborn lambs in neonatal period and its progression

**DOI:** 10.1186/s12899-016-0020-5

**Published:** 2016-01-19

**Authors:** Karoline Koether, Carla Maria Vela Ulian, Maria Lucia Gomes Lourenço, Renato Souza Gonçalves, Mateus José Sudano, Raíssa Karolliny Salgueiro Cruz, Naiana da Silva Branchini, Angélica Alfonso, Simone Biagio Chiacchio

**Affiliations:** 1 Department of Clinical Veterinary of School of Veterinary Medicine and Animal Science, State University of São Paulo, (UNESP), 18619-970 Botucatu, São Paulo Brazil; 2 Department of Medical Clinical, Botucatu Medical School, 18618-970 Botucatu, Brazil; 3 Laboratory of Genetics and Animal Breeding, Federal University of Pampa, 97508-000 Uruguaiana, RS Brazil

**Keywords:** Electrocardiogram, Newborn, Electrophysiology, Ovine, Cardiac development

## Abstract

**Background:**

Veterinary cardiology, especially electrocardiography, has shown major advancements for all animal species. Consequently, the number of ovine species used as experimental animals has increased to date. Few studies have been published on ovine systematic electrocardiography, particularly with respect to lamb physiology and neonatology. This study aimed to standardize the values of normal waves, complexes, and intervals of the electrocardiogram (ECG) in clinically Bergamasca healthy neonatal lambs, used as experimental animals. Serial computerized electrocardiography was performed in 10 male and 12 female neonates on the 1st, 7th, 14th, 21st, 28th, and 35th days of age. The following parameters were analyzed: heart rate and rhythm, duration and amplitude of waves, duration of intervals, and heart electrical axis.

**Results:**

During the first 35 days of life, (1) the sinusal heart rhythm was predominant, (2) there was a progressive decrease in the heart rate and R and T wave amplitude, and (3) a progressive increase in the PR, QT, and RR intervals. Finally, we confirmed that various components of neonatal evolution were more discernible in the augmented unipolar leads (aVF), which we recommend should be preferentially used in future studies. No significant statistical alterations were observed between males and females in relation to the analyzed parameters.

**Conclusions:**

The information assimilated in this study is anticipated to enhance the diagnosis of multiple congenital heart defects in Bergamasca lambs and could be implemented in studies that use ovine species as experimental models.

## Background

The way in which the electrocardiogram changes from birth to adulthood is known for humans [1]. According to the Brazilian Society of Cardiology [2], the most complete tabulation was drafted by Davignon et al. [3, 4], which represents an invaluable aid in the interpretation of the electrocardiogram of children. The electrocardiographic study showed frequent changes during the first year of life, particularly during the neonatal period (from the 1st to the 30th day), reflecting the anatomical and physiological changes that occur in preterm and/or shortly after birth [5, 6].

The cardiac physiology of lambs at birth differs to that of adults, with the electrocardiographic tracing of neonates being influenced by various parameters. Examples include blood pressure [5–7], peripheral vascular resistance [6], cardiac output [8], the relationship between the right and left ventricular masses [9, 10], anatomical conformation [11, 12], and autonomic innervations [7, 13–15]. Most electrocardiographic studies of sheep have been conducted on adult animals or fetuses, with descriptions about neonates remaining limited in the published literature [15].

The circulatory physiology of neonates and young animals differs to that of adults; thus, it is important to evaluate the cardiovascular system to develop knowledge about structural and functional differences that occur in young animals, such as lambs, in the period following birth [15, 16]. During the first month of life, an acute phase of circulatory and respiratory adaptation occurs in the neonates of different species. At this time, there is more variation in circulatory and respiratory parameters compared to adults, particularly during the period immediately after birth, because of rapid but varied hemodynamic changes [15, 17, 18]. This phenomenon arises because the electrocardiogram of a newborn reflects the hemodynamic repercussions over the heart on intrauterine life, with changes arising during the transition from fetus to neonate [4].

This study aimed to standardize and characterize the electrocardiogram of healthy newborn Bergamasca lambs from the 1st to 35^t^h day of age, by evaluating the electrocardiographic evolution that occurs during this period.

## Results

Gender had no effect on any of the studied parameters; however, the electrocardiographic events of lambs exhibited age-related changes (P < 0.05). The electrocardiographic parameters changed over time. Of all the analyzed leads, the aVF lead proved to be the most sensitive at detecting age-related changes on the electrocardiographic tracing. The effect of age on the electrocardiographic parameters was better visualized in the derivation aVF, where the values for the duration (P, PR, QRS, QT, and T) and amplitude (R and T) of the electrocardiographic waves varied significantly (P < 0.05) at the different analyzed times (Tables 1 and 2).

**Table 1:** The duration of ECG parameters in neonatal lambs

Lead	Age (days)	P (s)	PR (s)	QRS (s)	QTc (s)	T (s)
I	Birth	0.043 ± 0.008	0.075 ± 0.015	0.036 ± 0.007^a*^	0.233 ± 0.023^a^	0.068 ± 0.014
	7	0.039 ± 0.008	0.070 ± 0.017	0.036 ± 0.007^a^	0.226 ± 0.033^a^	0.068 ± 0.019
	14	0.037 ± 0.007	0.067 ± 0.012	0.035 ± 0.009^a^	0.236 ± 0.020^ab^	0.060 ± 0.014
	21	0.037 ± 0.006	0.070 ± 0.0086	0.040 ± 0.008^a^	0.249 ± 0.020^bc^	0.066 ± 0.011
	28	0.039 ± 0.006	0.072 ± 0.014	0.035 ± 0.006^a^	0.249 ± 0.019^c^	0.068 ± 0.007
	35	0.039 ± 0.007	0.077 ± 0.017	0.042 ± 0.009^b^	0.255 ± 0.027^d^	0.069 ± 0.014
II	Birth	0.040 ± 0.008	0.070 ± 0.016	0.039 ± 0.006	0.234 ± 0.022^ab^	0.076 ± 0.012
	7	0.039 ± 0.007	0.074 ± 0.022	0.040 ± 0.011	0.226 ± 0.020 ^b^	0.068 ± 0.015
	14	0.039 ± 0.008	0.066 ± 0.008	0.039 ± 0.009	0.230 ± 0.022^b^	0.060 ± 0.015
	21	0.040 ± 0.007	0.073 ± 0.009	0.052 ± 0.049	0.248 ± 0.017^ac^	0 066 ± 0.010
	28	0.041 ± 0.006	0.074 ± 0.009	0.042 ± 0.008	0.255 ± 0.019^c^	0.070 ± 0.010
	35	0.040 ± 0.005	0.075 ± 0.011	0.042 ± 0.008	0.263 ± 0.027^c^	0.067 ± 0.011
III	Birth	0.040 ± 0.006	0.069 ± 0.014^a^	0.036 ± 0.008	0.231 ± 0.022^ab^	0.068 ± 0.014^ac^
	7	0.037 ± 0.008	0.067 ± 0.015^a^	0.038 ± 0.007	0.217 ± 0.025^b^	0.059 ± 0.014^b^
	14	0.038 ± 0.008	0.066 ± 0.011^a^	0.037 ± 0.009	0.233 ± 0.017^bc^	0.060 ± 0.011b^c^
	21	0.041 ± 0.007	0.073 ± 0.010^ab^	0.037 ± 0.012	0.246 ± 0.01^bd^	0.061 ± 0.011^bc^
	28	0.040 ± 0.006	0.070 ± 0.010^a^	0.042 ± 0.013	0.252 ± 0.015^d^	0.066 ± 0.012^abc^
	35	0.042 ± 0.006	0.079 ± 0.014^b^	0.041 ± 0.008	0.260 ± 0.030^d^	0.067 ± 0.012^c^
aVR	Birth	0.039 ± 0.007	0.066 ± 0.012^a^	0.035 ± 0.007	0.236 ± 0.023^ab^	0.074 ± 0.011^a^
	7	0.035 ± 0.005	0.067 ± 0.010^a^	0.035 ± 0.008	0.222 ± 0.026^b^	0.065 ± 0.017^b^
	14	0.037 ± 0.009	0.066 ± 0.009^a^	0.035 ± 0.007	0.230 ± 0.023^ab^	0.058 ± 0.012^c^
	21	0.035 ± 0.005	0.070 ± 0.007^ab^	0.037 ± 0.007	0.245 ± 0.020^ac^	0.067 ± 0.013^b^
	28	0.040 ± 0.005	0.075 ± 0.015^b^	0.035 ± 0.010	0.252 ± 0.017^c^	0.069 ± 0.008^b^
	35	0.037 ± 0.005	0.075 ± 0.011^b^	0.035 ± 0.007	0.261 ± 0.023^c^	0.072 ± 0.010^b^
aVL	Birth	0.040 ± 0.009^ac^	0.069 ± 0.017^ab^	0.047 ± 0.063	0.234 ± 0.027^ab^	0.059 ± 0.010^ab^
	7	0.035 ± 0.007^b^	0.067 ± 0.013^a^	0.039 ± 0.009	0.219 ± 0.017^b^	0.060 ± 0.011^abc^
	14	0.035 ± 0.007^bc^	0.065 ± 0.0085^a^	0.038 ± 0.006	0.235 ± 0.022^bc^	0.056 ± 0.012^a^
	21	0.039 ± 0.007^c^	0.077 ± 0.017^bc^	0.040 ± 0.01	0.243 ± 0.015^ac^	0.065 ± 0.013^b^
	28	0.040 ± 0.008^cd^	0.075 ± 0.013^bc^	0.037 ± 0.007	0.251 ± 0.018^cd^	0.062 ± 0.011^abc^
	35	0.041 ± 0.009^d^	0.080 ± 0.016^c^	0.041 ± 0.009	0.261 ± 0.025^d^	0.067 ± 0.011^c^
aVF	Birth	0.041 ± 0.005^ac^	0.070 ± 0.016^abcd^	0.039 ± 0.007^a^	0.235 ± 0.023^ab^	0.072 ± 0.012^ac^
	7	0.037 ± 0.006^b^	0.068 ± 0.015^abc^	0.036 ± 0.006^a^	0.219 ± 0.019^b^	0.062 ± 0.016^bc^
	14	0.037 ± 0.006^b^	0.066 ± 0.008^b^	0.040 ± 0.010^ab^	0.234 ± 0.020^ab^	0.060 ± 0.013^b^
	21	0.042 ± 0.005^c^	0.074 ± 0.010^cd^	0.040 ± 0.008^ab^	0.245 ± 0.018^ac^	0.065 ± 0.007^bc^
	28	0.043 ± 0.005^ac^	0.075 ± 0.007^d^	0.044 ± 0.008^b^	0.251 ± 0.018 ^ad^	0.063 ± 0.010^bc^
	35	0.042 ± 0.006^ac^	0.075 ± 0.013^d^	0.040 ± 0.008^ab^	0.266 ± 0.027 ^d^	0.068 ± 0.011^c^
BA	Birth	0.055 ± 0.033	0.075 ± 0.011	0.042 ± 0.006	0.233 ± 0.021^ab^	0.175 ± 0.0267^ab^
	7	0.045 ± 0.009	0.078 ± 0.010	0.042 ± 0.007	0.222 ± 0.021^a^	0.162 ± 0.0259^ab^
	14	0.047 ± 0.008	0.075 ± 0.009	0.042 ± 0.009	0.225 ± 0.016^a^	0.167 ± 0.0189^a^
	21	0.046 ± 0.007	0.080 ± 0.008	0.042 ± 0.007	0.239 ± 0.015^ac^	0.184 ± 0.0204^bc^
	28	0.046 ± 0.008	0.082 ± 0.012	0.043 ± 0.005	0.247 ± 0.017^cd^	0.194 ± 0.0194^c^
	35	0.046 ± 0.005	0.082 ± 0.011	0.042 ± 0.006	0.258 ± 0.028^d^	0.210 ± 0.0343^d^

(*) Superscript letters in the same column are significantly different (*P* < 0.05) differences between time points (days of age). Values are expressed as mean ± SD

*S: * seconds, *QTc: corrected QT interval*

**Table 2:** Amplitude of ECG parameters in neonatal lambs

Lead	Age (days)	P (mV)	R (mV)	S (mV)	T (mV)
I	Birth	0.097 ± 0.036	0.140 ± 0.168	0.085 ± 0.102	0.246 ± 0.159^a*^
	7	0.105 ± 0.038	0.123 ± 0.120	0.115 ± 0.164	−0.046 ± 0.213^b^
	14	0.103 ± 0.023	0.081 ± 0.099	0.114 ± 0.104	0.024 ± 0.128^c^
	21	0.104 ± 0.027	0.105 ± 0.087	0.085 ± 0.127	0.057 ± 0.140^c^
	28	0.111 ± 0.035	0.103 ± 0.110	0.113 ± 0.108	0.178 ± 0.115^d^
	35	0.109 ± 0.028	0.076 ± 0.091	0.147 ± 0.134	0.166 ± 0.124^d^
II	Birth	0.133 ± 0.054	0.225 ± 0.159^a^	0.073 ± 0.097	0.462 ± 0.160^a^
	7	0.137 ± 0.044	0.128 ± 0.120^b^	0.107 ± 0.109	0.221 ± 0.119^b^
	14	0.130 ± 0.035	0.138 ± 0.105^b^	0.091 ± 0.103	0.159 ± 0.086^bc^
	21	0.132 ± 0.033	0.114 ± 0.096^b^	0.093 ± 0.109	0.152 ± 0.138^c^
	28	0.113 ± 0.034	0.135 ± 0.098^b^	0.100 ± 0.125	0.256 ± 0.152^d^
	35	0.125 ± 0.030	0.115 ± 0.094^b^	0.121 ± 0.148	0.295 ± 0.144^d^
III	Birth	0.109 ± 0.036^a^	0.175 ± 0.119	0.066 ± 0.100	0.244 ± 0.168^a^
	7	0.107 ± 0.030^a^	0.179 ± 0.121	0.078 ± 0.113	−0.020 ± 0.175^b^
	14	0.106 ± 0.020^a^	0.166 ± 0.107	0.056 ± 0.095	0.046 ± 0.175^bd^
	21	0.093 ± 0.017^a^	0.149 ± 0.113	0.075 ± 0.120	0.136 ± 0.126^ce^
	28	0.078 ± 0.053^b^	0.145 ± 0.112	0.070 ± 0.097	0.116 ± 0.139^de^
	35	0.096 ± 0.024^a^	0.140 ± 0.131	0.100 ± 0.159	0.159 ± 0.139^e^
aVR	Birth	−0.122 ± 0.054	0.110 ± 0.0798	0.135 ± 0.175^a^	−0.311 ± 0.205^a^
	7	−0.101 ± 0.056	0.133 ± 0.120	0.068 ± 0.105^b^	0.054 ± 0.211^b^
	14	−0.115 ± 0.032	0.120 ± 0.0839	0.040 ± 0.080^b^	0.015 ± 0.154^bc^
	21	−0.109 ± 0.028	0.0995 ± 0.103	0.067 ± 0.078^b^	−0.069 ± 0.152^c^
	28	−0.111 ± 0.030	0.120 ± 0.113	0.074 ± 0.109^b^	−0.203 ± 0.157^d^
	35	−0.114 ± 0.026	0.141 ± 0.109	0.040 ± 0.066^b^	−0.183 ± 0.170^d^
aVL	Birth	−0.047 ± 0.067^a^	0.110 ± 0.120	0.102 ± 0.115	0.057 ± 0.146
	7	−0.025 ± 0.088^b^	0.109 ± 0.096	0.114 ± 0.151	−0.013 ± 0.147
	14	−0.016 ± 0.085^b^	0.058 ± 0.091	0.126 ± 0.101	0.010 ± 0.127
	21	−0.070 ± 0.054^c^	0.080 ± 0.099	0.092 ± 0.122	0.028 ± 0.148
	28	−0.089 ± 0.019^c^	0.074 ± 0.106	0.102 ± 0.108	0.071 ± 0.097
	35	−0.076 ± 0.048^c^	0.068 ± 0.105	0.135 ± 0.121	0.072 ± 0.150
aVF	Birth	0.118 ± 0.037	0.189 ± 0.118^a^	0.063 ± 0.105	0.315 ± 0.200^a^
	7	0.112 ± 0.029	0.149 ± 0.092^b^	0.069 ± 0.094	−0.042 ± 0.224^b^
	14	0.109 ± 0.023	0.125 ± 0.093^b^	0.069 ± 0.085	0.048 ± 0.153^c^
	21	0.106 ± 0.024	0.128 ± 0.098^b^	0.072 ± 0.108	0.153 ± 0.102^d^
	28	0.099 ± 0.025	0.132 ± 0.107^b^	0.073 ± 0.101	0.182 ± 0.143^d^
	35	0.105 ± 0.0215	0.112 ± 0.106^b^	0.094 ± 0.139	0.187 ± 0.132^d^
BA	Birth	0.232 ± 0.066^a^	0.034 ± 0.046	0.586 ± 0.229	0.452 ± 0.292^a^
	7	0.217 ± 0.055^a^	0.036 ± 0.047	0.443 ± 0.156	0.284 ± 0.146^b^
	14	0.223 ± 0.045^ab^	0.014 ± 0.033	0.462 ± 0.140	0.267 ± 0.131^b^
	21	0.200 ± 0.043^bc^	0.024 ± 0.056	0.405 ± 0.092	0.315 ± 0.157^a^
	28	0.179 ± 0.030^c^	0.029 ± 0.044	0.401 ± 0.131	0.328 ± 0.160^a^
	35	0.175 ± 0.038^c^	0.025 ± 0.050	0.448 ± 0.173	0.332 ± 0.152^a^

(*) Superscript letters in the same column are significantly different (P < 0.05) differences between time points (days of age). Values are expressed as mean ± SD. mV: milivolt

There were significant differences in the median HR during the first 35 days of age. During the first 24 h after birth, the median heart rate was 91.955 ± 42.176 beats per minute (range: 290–121 bpm). During the first two postnatal weeks, the median heart rate increased to 214.955 ± 54.582 bpm (range: 305–122 bpm) and 195.5 ± 37.744 bpm (range: 260–108 bpm), respectively. With each subsequent week, this parameter significantly declined (P < 0.001), with the lowest value being obtained at 35 days of age, 145.636 ± 37.803 bpm (range: 260–92 bpm) (Table 3).

**Table 3:** Heart rate, RR interval, and electrical axis of neonatal lambs

Age (days)	Body weight (Kg)	BSA (m^2^)	Heart rate (bpm)	RR interval (ms)	Axis
Birth	3.88 ± 0.88^a^	0.0025 ± 0.00037^a^	191.95 ± 42.17^a*^	334.86 ± 69.15^a^	29.63 ± 108.50°
	(2.40–5.60)	(0.0018–0.0032)	(193–160)	(490–230)	
7	5.30 ± 1.31^b^	0.0030 ± 0.00050^b^	214.95 ± 54.58^b^	297.04 ± 69.12^a^	5.31 ± 110.39°
	(3.00–7.60)	(0.0021–0.0039)	(305–122)	(413–197)	
14	6.84 ± 1.78^c^	0.0036 ± 0.00062^c^	195.50 ± 37.74^ab^	305.90 ± 57.22^a^	−18.86 ± 115.31°
	(3.40–10.00)	(0.0022–0.0047)	(260–108)	(480–223)	
21	8.17 ± 2.05^d^	0.0041 ± 0.00067^d^	173.04 ± 30.72^c^	361.18 ± 56.17^d^	−8.45 ± 113.61°
	(4.60–12.00)	(0.0028–0.0053)	(250–110)	(467–270)	
28	9.50 ± 2.45^e^	0.0045 ± 0.00077^e^	158.81 ± 22.14^cd^	379.81 ± 44.88^b^	−37.77 ± 113.36°
	(5.55 ± 13.20)	(0.0031–0.0056)	(211–116)	(467–320)	
35	10.78 ± 2.91^f^	0.0049 ± 0.00088^f^	145.63 ± 37.80^d^	441.63 ± 115.28^c^	−29.63 ± 117.39°
	(5.50 ± 15.10)	(0.0031–0.0062)	(260–92)	(230–423)	

(*) Superscript letters in the same column are significantly different (P < 0.05) between time points (days of age)*.* Values are expressed as mean ± SD. *BSA:* body superface area

The correlation of several parameters related to body surface area and the body weight in neonate lambs are shown in the Table 4.

**Table 4:** Correlation of several parameters with the body surface area and the body weight in neonate lambs

Parameters	BSA			Body weight		
	R	95 % CI	*P*	R	95 % CI	*P*
Heart rate	−0.2139	−0.3713, −0.04466	0.0138	−0.2170	−0.3741, −0.04787	0.0124
P (ms)	0.03876	−0.1380, 0.2132	0.6590	0.03876	−0.1380, 0.2132	0.6590
P (mV)	0.05074	−0.1262, 0.2246	0.5634	0.05074	−0.1262, 0.2246	0.5634
PR (ms)	−0.03408	−0.2087, 0.1426	0.6981	−0.03408	−0.2087, 0.1426	0.6981
QRS (ms)	−0.02338	−0.1984, 0.1531	0.7902	−0.02338	−0.1984, 0.1531	0.7902
R (mV)	−0.3565	−0.5009, −0.1927	<0.0001	−0.3637	−0.5033, −0.2055	<0.0001
QTc (mV)	0.2523	0.08507, 0.4057	0.0035	0.2512	0.08394, 0.4048	0.0037
T (ms)	−0.09783	−0.2691, 0.07940	0.2644	−0.09783	−0.2691, 0.07940	0.2644
T (mV)	0.1863	0.01079, 0.3507	0.0324	0.1863	0.01079, 0.3507	0.0324
Axis (°)	−0.09967	−0.2708, 0.07755	0.2555	−0.09967	−0.2708, 0.07755	0.2555

*BSA:* body surface area, *CI:* confidence interval,Level of significance: P<0,05

When a gradual increase in RR interval was observed, a parallel decrease in HR was also documented. These parameters, RR and HR, significantly changed as the lambs physically matured. The average RR interval gradually increased from the 21st day after birth (361.182 ± 56.174 ms), reaching a maximum at 35 days of after birth (441.636 ± 115.282 ms) (P < 0.001).

A negative correlation was recorded between the FC and RR interval every week after birth, except at 28 days of age (r = −0.409; P = 0.058); specifically, birth (r = −0.769; P < 0.001); 7 days (r = −0.798; P < 0.001); 14 days (r = −0.475; P = 0.0255); 21 days (r = −0.652; P < 0,001); and 35 days (r = −0.653; P < 0.001).

The QT interval duration had significant more expressive increase from 21st day of age (0.248 ± 0.017 s), in all analyzed leads, when compared to the birth and the first two weeks of age (0.230 ± 0.022 s), describing its longest value at 35th day (0.263 ± 0.027 s) (P <0.001). The interval showed negative correlation with heart rate (r = −0.7560; P < 0.0001), and positive correlation with age (r = 0.4770; P < 0.0001).

The P wave morphology were mainly positive in I, II, III and aVF leads, predominantly negative in aVR and variable in aVL lead, which showed negative configuration in 28.78 % of 132 electrocardiographic tracings especially on first three weeks of age, thenceforth predominating positive in 71.21 %, until 35 days of age.

A wide variety of configurations of the QRS complex was observed during the study in the leads of the frontal plane. There was a 77.72 % of QRS complexes of type rS and qR in I and aVF lead respectively during the first week of age; 54.54 % qR in I and rS in aVF lead on 7 days; 63.63 % of rS in I and aVF on 14 days; 59.09 % qR in I and rS in aVF lead on 21 days; 68.18 and 72, 72 % of rS in I and aVF on 28 and 35 days respectively. It was observed an evolution of the complex during the study, thus obtaining a greater percentage of low-voltage and complexes with standard qs, qr and rs.

The electrocardiographic T wave evolutionary behavior revealed differentiated in duration and amplitude throughout the weeks. The amplitude (I, II, III, aVR, aVF and base-apex) decreased significantly on 7th and 14th day (0.048 ± 0.153 mV) when compared to birth (0.315 ± 0.200 mV) (P = <0.001). The amplitude of T increased from 21st day (0.153 ± 0.102 mV), thus remaining until the 35 th day (Table 5).

**Table 5:** Different morphologies of T waves, observed in neonate lambs in different leads

Lead	Types of waves	Number of animals (%)
I	Positive (+) Negative (−) Biphase (− +)	96 (72,72 %) 31 (23,48 %) 5 (3,78 %)
II	Positive (+) Negative (−) Biphase (− +)	108 (81,81 %) 21 (15,90 %) 3 (2,27 %)
III	Positive (+) Negative (−) Biphase (− +)	98 (74,24 %) 30 (22,72 %) 4 (3,03 %)
aVR	Positive (+) Negative (−) Biphase (− +)	35 (26,51 %) 94 (71,21 %) 3 (2,27 %)
aVL	Positive (+) Negative (−) Biphase (− +)	86 (65,15 %) 45 (34,09 %) 1 (0,75 %)
aVF	Positive (+) Negative (−) Biphase (− +)	105 (79,54 %) 24 (18,18 %) 3 (2,27 %)
BA	Positive (+) Negative (−) Biphase (− +)	132 (100 %) 0 (0 %) 0 (0 %)

In contrast, the duration of the T wave (III, aVR, aVL, aVF and base-apex) decreased on the first three weeks of age, thenceforth beginning to increase and remaining until the 35 days of age.

The distribution of the heart rhythm in serial ECGs of neonatal lambs at 35 days of age was 38.63, 31.06, and 26.51 % for the sinus tachycardia, sinus arrhythmia, and sinus rhythm, respectively. Disturbances in the formation of the electrical impulse found during the study occurred in the form of atrial and ventricular premature complexes (2.25 and 0.75 %, respectively), in addition to disturbances in impulse conduction due to sinoatrial block (0.75 %).

The mean interval and standard deviation of P, R, S T wave durations, PR and QT intervals, the QRS complex, and the mean amplitude of P, Q, R S, and T waves are shown in Tables 1 and 2. The evolutionary behavior of electrocardiographic waves was only significant in certain leads, despite being similar in all leads.

There were significant differences in the QTc interval dispersions during the first 35 days of age. During the first 24 h of life, the median was 0.086 ± 0.014 s. In the first seven days, this value increased significantly, and decreasing at 14th, reaching its shortest length at 21st day (0.066 ± 0.006 s). In the following weeks, this parameter turned to increase, reaching its maximum at 35th (0.112 ± 0.004 s). The maximum P-wave and PWD values were not statistically different during all period evaluated. The longest value of PWD was at birth (0.045 ± 0.055 s), and the shortest were at 28th (0.022 ± 0.004 s) and 35th (0.022 ± 0.007 s) days. Maximum P-wave duration showed its longest duration at birth (0.075 ± 0.055 s) and shortest at seventh (0.051 ± 0.006 s) and 28th (0.052 ± 0.004 s) days. The mean interval and standard deviation of P wave and QTc interval dispersions in neonatal lambs are shown in Table 6.

**Table 6:** P wave and QTc interval dispersions in neonatal lambs

	Birth	7 days	14 days	21 days	28 days	35 days	P value
P min	0.030 ± 0.00	0.027 ± 0.004	0.025 ± 0.005	0.028 ± 0.003	0.030 ± 0.0	0.031 ± 0.003	0.0796
P max	0.075 ± 0.055	0.051 ± 0.006	0.055 ± 0.007	0.055 ± 0.005	0.052 ± 0.004	0.054 ± 0.007	0.3487
PWD	0.045 ± 0.055	0.024 ± 0.005	0.030 ± 0.008	0.027 ± 0.004	0.022 ± 0.004	0.022 ± 0.007	0.4119
QTc min	0.188 ± 0.009^ab^	0.179 ± 0.020^a^	0.187 ± 0.010^ab^	0.207 ± 0.009^bc^	0.211 ± 0.010^c^	0.195 ± 0.00^abc^	0.0004
QTc max	0.274 ± 0.007^ab^	0.279 ± 0.022^ab^	0.274 ± 0.007^ab^	0.274 ± 0.007^ab^	0.283 ± 0.010^ab^	0.308 ± 0.007^c^	<0.0001
QTcID	0.086 ± 0.014^ab^	0.099 ± 0.029^ac^	0.086 ± 0.011^ab^	0.066 ± 0.006^b^	0.072 ± 0.010^b^	0.112 ± 0.004^c^	<0.0001

*PWD* P wave dispersion, *P max* maximum P wave duration, *Pmin* minimum P wave duration

*QTcD* QT correted interval dispersion, *QTc max* maximum QTc interval duration, Q*Tc min* minimum *QTc* interval duration. Superscript letters in the same line are significantly different (P < 0.05). Values are expressed as mean ± SD. Results expressed as mean ± standard deviation

Only six animals were obtained from twin pregnancies, thus it was not possible to perform statistical analysis to detect possible differences between the electrocardiographic parameters of lambs from twin and singleton pregnancy.

## Discussion

Published literature about neonate lamb electrocardiography remains limited; hence, it was difficult to compare the results of this study with previous publications. Some of the values obtained from the frontal plane and base-apex leads have not been previously collected for the studied parameters and have not been previously determined for this age group, further confirming the lack of references on this subject.

There was no information available in the published literature about the relationship between the sex of animals and cardiac physiology for neonates. In the current study, we did not observe any influence of gender on any of the evaluated parameters, with no statistically significant differences being obtained (P < 0.05) between males and females. A study on rodents detected a difference in the heart rate between the sexes immediately after weaning, with it decreasing in females and increasing in males. Other electrocardiographic parameters, such as PR and QT intervals, were also shown to differ between male and female rodent offspring in the same study [19]. Electrocardiographic research is necessary in ovine species to determine whether the endocrine system acts on the cardiovascular system of various age groups, especially in relation to sex hormones [20].

By positioning animals in a dextro-lateral position, it was possible to monitor the ECG pattern with standard bipolar leads by using de Einthoven’s triangle and identify the electrocardiographic components that were discernible from each lead, as described for goats [21, 22] and for sheep [23, 24]. In previous studies, although changes in body position influenced the QRS in the current study, electrical axis, and P and T waves showed little change, supporting previous research [11]. Broad variability was detected in the electrocardiographic tracings at all times, which is an inherent characteristic of ovine species. However, it was still possible to analyze the electrocardiographs to detect cardiac arrhythmias, supporting the findings of previous studies [11, 25, 26]. Previous studies indicated that anatomical differences between the hearts of carnivores and sheep do not favors the use of the frontal plane for ECGs. Examples include a 90° inclination from the axis in the positioning of the heart in the thorax in sheep, in addition to the process of ventricular depolarization in sheep. Such anatomical differences generate wide variability in the ECG, with small amplitude waves and complexes being obtained, making it difficult to visualize. In the current study, as lambs physically matured, the electrocardiographic tracings changed, with the aVF lead being the most sensitive of all frontal plane and base-apex leads. It is likely that the anatomical features of the lamb heart favors the classical method of using the frontal plane during the neonatal period; however, for mature sheep, the sagittal axis becomes the most viable method, as suggested by literature [11, 26].

Time was observed to have a significant effect on the heart rate and the RR interval in this study, whereby heart rate decreased and the of RR interval increased from birth to 35 days of age. We observed a negative correlation between age and heart rate (r = −0.46; P < 0.0001), and a positive correlation between age and the RR interval (r = 0.47; P < 0.0001). Our results support those previous [27, 28]. Immediately after birth, the neonate has low pressure, low systolic volume, and low peripheral vascular resistance. To maintain peripheral perfusion, the neonate must maintain a higher heart rate, cardiac output, plasma volume, and central venous pressure [29]. In addition, the heart rate of the fetus and the newborn is quick and relatively unstable. This phenomenon may be explained, in part, by the immaturity of the autonomic nervous system, with sympathetic innervations forming later in development; hence, neural control is predominantly cholinergic at birth [15, 30, 31].

The predominant heart rate observed in this study was sinus tachycardia, followed by sinus arrhythmia. Sinus tachycardia is characterized by regular sinus rhythm, with the heart rate being higher compared to the reference limits established for a given species, or more suitable for certain behaviors or age groups. Sinus tachycardia of adult sheep is represented by a regular rhythm from the sinus node, exceeding 115 bpm [32], however, the values for determining sinus tachycardia in neonatal lambs have not been previously established [16]. In our study, sinus tachycardia was identified from the average heart rate of the age group in question. Lambs were considered to be in tachycardia when the average heart rate was above the median for a given age group. Although this classification was arbitrary, the reference limits for adults could not be used because of the comparatively high heart rates of lambs during the neonatal period; consequently, physiological rhythm would have been considered just sinusal. Behavioral circumstances and stress caused by carrying out the examination are also likely to cause an increase in lamb heart rates [20]. The presence of sinus arrhythmia reflects the immaturity of the autonomic nervous system and the prevalence of parasympathetic innervations at birth [33].

Sheep are often used as experimental animal models to study cardiac diseases, such as atrial fibrillation (AF) [34, 35]. Butters et al. [36] created a novel biophysically detailed computational model of the three-dimensional sheep atria, and it was found that the electrical heterogeneity and the anisotropic property in the atrial fiber structure that occurs in the specie plays an important role on development and sustainability of this arrhythmia. Zhao et al. [37] observed similar myo-bundle structure in the human and sheep atria, for example in Bachmann’s bundle, atrial septum, pectinate muscles, superior vena cava and septo-pulmonary bundle. The authors also confirmed that the preferential propagation pathways of the activation sequence in both atrial models is qualitatively similar, largely due to the domination of the major muscle bundles.

In our study, among supraventricular arrhythmias, the premature atrial contractions were observed in one pair of twin lambs and persisted at different stages of development during the study period. This type of disturbance is observed in congenital heart defects, such as the patent ductus arteriosus [16, 38]. Thus, considering the similarity of heart between human and sheep, as well as its electrical heterogeneity and structural anisotropy propitious to supraventricular arrhythmias, we can say that researches about arrhythmias in newborns sheep can contribute in human neonatology.

The allometric relationship between size and/or body weight and heart rate have been described during years as inversely proportional between the various domestic species, this relation is higher in species like small rodents (500 – 700 bpm) and smaller in whales (20 bpm) [39, 40].

Milnor [41] described an inverse correlation between heart rate and body weight through measurement of aortic impedance, which is defined as the load imposed on the ventricle by the properties of the arterial tree through minimum records pulsatile pressure and flow in the aorta ascendant.

Premature atrial contractions were observed in one pair of twin lambs and persisted at different stages of development during the study period. This type of disturbance to the formation of rhythm is observed in congenital heart defects, such as the patent ductus arteriosus [16, 38]. Isolated premature ventricular contractions were documented in only one lamb until 28 days of age. This phenomenon may occur in normal hearts with no apparent cause. After the clinical examination of this individual, no further clinical signs were detected. Sinoatrial block was detected in two lambs soon after birth. This type of disorder may arise secondarily to changes in the autonomic tonus and hypoxia following birth [16, 38].

Electrocardiogram characteristics should be considered in relation to age [4]. Our results showed frequent changes in the ECG, particularly during the neonatal period. These variations reflect both the anatomical and physiological changes that occur shortly after birth. Therefore, it is essential to obtain baseline average values for the waves, ranges, and complexes to accurately interpret the electrocardiograms of lambs. The values of certain parameters obtained from the six leads placed in the frontal plane and base-apex in this study were not available in the published literature for this age group of the Bergamasca breed. In general, our results differed to those obtained for adults, but were similar to those described by Tovar et al. [42–45].

The length and amplitude of the wave P values throughout the neonatal period differed to those described for adult animals [23]. The P wave of newborn lambs was high, sharp, and asymmetrical. These characteristics were related to physiological tachycardia, particularly during the first 2 weeks after birth (at 7 and 14 days). The decrease in P wave duration coincided with an observed elevation in heart rate. During neonatal development, P waves gradually became shorter and wider because of a normal increase in atrial size [30]. As the lambs grew, the amplitude of the P wave declined, with values declining until they became similar to those described for adults. Adults have the lowest P wave range because of their larger body size, which increases the distance between the electrode and the focus of atrial depolarization, promoting a lower voltage [44, 45].

In the current study, the PR interval increased with age and was inversely correlated with the heart rate. These values differed to those described for adults [23, 25], remaining inferior to adult values until the lambs were 35 days of age. In general, the PR value is quite short at birth and during the first week of life, gradually lengthening until 5 years of age, which is similar to the total PR interval described for human infants and children [30]. The duration of the PR interval reflects the time between atrial and ventricular depolarization. Therefore, this parameter serves as an important indicator of heart block and cardiac conduction abnormalities [19]. Hence, the detection of a change in the relationship between the PR interval and fetal heart rate could potentially be used to detect fetal compromise in sheep, further highlighting the importance of age-matched control data [46].

A gradual increase in the duration of ventricular depolarization (QRS complex) coinciding with the growth and body development, indicates a possible increase in cardiac muscle mass. However, the values obtained in this study were very similar to those described for adults. According to Tovar et al. [43, 44], the QRS complex increases from birth until 2 months of age, with no significant difference being obtained between 2 months and 1 year of age. In the current study, QRS was negatively correlated with cardiac frequency during the neonatal period (r = − 0.347; P < 0.001), but did not influence the duration of the cardiac cycle (RR interval). The electrocardiographic characteristics of right ventricular hypertrophy of newborns include physiological changes in the amplitude of the R and S waves and the R/S ratio [17]. However, these characteristics could not be identified in our study; therefore, it is likely that the QRS duration increased over the initial 3 weeks of life to occur predominantly in the left ventricle or left ventricular hypertrophy. A decrease in QRS complex amplitude with advancing age was also observed by Tovar et al. [42, 43] which was explained by an increased degree of cancellation promoted by the development of the Purkinge network during the neonatal period. With the animal growth and consequents gains of body surface area and body weight, it also occurs the development of cardiac mass, and decreases in R-wave amplitude is related to the maturation of the His bundle and Purkinje cells, carrying to synchronous depolarization [38, 47].

Ventricular repolarization is influenced by maturity, with a significant decrease in amplitude being recorded within 2 weeks of birth. According to Tovar et al. [42, 43], the most significant decrease in amplitude occurs within the first 4 days of birth. The high polarity of the T wave immediately after birth is probably the result of respiratory abnormalities caused by the birthing process and the immaturity of the autonomic tonus. The prominence of the T wave in lambs signifies a rapid and major contraction of the basal part of ventricles, followed by the repolarization phenomena of the ventricles [24].

The wave and complex morphology was quite variable throughout the neonatal period. Various QRS patterns were found; however, no specific pattern predominated for a particular week of growth, contrary to that literature described [42–45, 48]. In general, lambs that exhibited a particular pattern at birth, maintained this pattern until 35 days of age, but at a reduced voltage. The observed values of the QTc interval significantly increased until 35 days of age in all leads, but remained below the established values for adults. We observed a directly proportional relationship among QTc intervals, the RR interval, and age. This relationship has been previously described by Tovar et al. [42, 43], who suggested that the duration of ventricular electrical systole greatly influences the duration of the cardiac cycle.

In a study by El-Gamal et al. [49], the authors observed prolongation of QTc interval in overweight patients, their result was in agreement with the findings of our study, in which a positive correlation was found between body weight and increase in length of QTc interval.

T wave polarity was also variable. Previous studies suggested that the polarity of the T wave is opposite to that of the QRS complex [23, 42, 43]; however, this phenomenon was not observed in our study, except on the base-apex lead. In the preceding studies, repolarization in sheep of 1 day to 3 months of age begins in the left apical regions, whereas it is initiated in the right ventricular epicardial area in 1-year-old sheep. The discrepancy between the preceding studies and our current results might be due to: (1) the low amplitude of the waves and difficulty in defining this parameter accurately [11]; (2) the technique used (conventional versus computerized electrocardiograph, respectively); (3) the immaturity of the conduction systems (bundle of His and Purkinge network in neonates); and (4) the broad variation in the direction of depolarization and repolarization, which is inherent in ovine species [48].

The anatomical position of the heart of humans and domestic mammals primarily differs in the way that the cardiac longitudinal axis tilts and the degree of rotation around this axis. For instance, part of the right ventricle extends to the left in ruminants. Therefore, ruminant hearts are rotated more to the left relative to human hearts [11]. Unlike caprine, equine, bovine, and porcine species, ovine species are categorized as type B; whereby, the electrical phenomena of ventricular activation are at the average end of the ventricular depolarization vector, with a base-apex, and a standing electric axis of −90° to −180° between the heart [42, 43]. During the neonatal period (from birth to 5 weeks of age), the electric axis of the lambs did not change significantly; however, it did differ to the values described for adults, ranging between +30° and −30°. We hypothesized that this deviation is caused by the right ventricle predominantly occurring on the left ventricular mass and by the positioning of the heart during the thorax in the neonatal period.

On a 12-lead ECG, maximum P-wave and P-wave dispersion (PWD) durations are used to determine whether the sinus impulse is distributed homogeneously and to evaluate the intra- and interatrial conduction times. Maximum P-wave is used as an indicator of interatrial conduction disorder, while PWD is used as a marker of regional differences in P-wave durations. Although findings have shown the P-wave duration to be important in a variety of clinical conditions, the most important of these is reported to be paroxysmal atrial fibrillation [50, 51]. The pressure, volume overload, electrolyte imbalance, or increase in sympathetic activity can increase the PWD. Duration of the P-wave reflects the activation of the atrial muscle and may depend on the mass of the tissue excited. Both P-wave duration and PWD are influenced by age due to decreased heart rate and increased weight and size of the heart [52–54]. In the current study, the decrease observed in these parameters over the weeks, is related to the electrolyte balance, the maturation of the ANS, decrease in heart rate and increase weight and body surface area that the neonates acquired with their development.

The QT dispersion (QTD) is an important electro physiologic marker that shows the differences in repolarization duration in a variety of electrocardiography leads and reflects the local differences in the recovery periods for ventricular myocardium. An increase in QTD can be associated with an increased risk of ventricular arrhythmias and sudden death, especially in patients with hypertrophic cardiomyopathy [55, 56].

According to Vialle et al. [57], QT-d and QTc-d were not related to age in children. The authors reported that QT dispersion did not change significantly with age and the mean value for the children aged from 5 days to 16 years was 36 ± 13.7 ms. Akyuz et al. [58] evaluated children with low birth weight and with normal birth weight. The mean value for QTc-d was 29 ms in all children. Although left ventricular mass index (LVMI) and QTc-d were normal values for the two groups, a positive relationship was seen between LVMI and QTc-d.

The literature [59], evaluating the effects of severe coarctation of the aorta (CoA) in a population of newborns (aged 45 ± 15 days), concluded that even when left ventricular hypertrophy is not present, the pressure overload after severe isolated CoA is responsible for an increased ventricular repolarization heterogeneity. Their data showed correct QT dispersion (QTcD) values of 23 ± 15 ms from the healthy control group, and 109.7 ± 43.4 ms from the Aortic coarctation group.

The value of the CoA group was similar than our group at 35° day, suggesting that in lambs, changes in pressure during the period of neonatal adaptation, can per se, influence regional dispersion of repolarization.

As a few number of twins were obtained this study (n = 6), it was not possible to conduct statistical procedure to detect possible difference from the electrocardiographic parameters of lambs coming from twin or singleton pregnancy. We believe that due to weight and body surface area of twin lambs, some parameters related to these variables, may differ from singletons animals, so more research is needed in order to obtain a better approach about this aspect in sheep.

## Conclusion

In conclusion, this study contributes novel information to the limited data currently available on the electrocardiography of neonate lambs. We confirm that the heart rate, rhythm, and amplitude, along with the duration of waves and intervals, morphology, and axis, of neonate lambs may be consistently assessed through a non-invasive method. For certain parameters, we were able to resolve developmental differences in ECG parameters in relation to neonatal development. We showed that, compared to adult sheep, lambs exhibited faster heart rates, longer PR intervals, and QT intervals, while the amplitude of the P and T waves decreased with increasing physical maturity. In addition, ventricular systole was correlated with heart cycle duration and electric axis deviation at various stages of postnatal lamb development.

## Methods

### Animal maintenance

A total of 22 Bergamasca lambs (10 males, 12 females) were enrolled in this study from birth to 35 days of age, born to them between August 2012 and December 2012. The animals were maintained in an intensive system and belong at the Sheep Breeding Farm to the School of Veterinary Medicine and Animal Science, in Botucatu, Brazil. The experiments were performed in accordance with Ethics Committee and was previously approved by the institutional committee on the ethics and animal welfare (The Ethics Committee on the Use of Animals - CEUA) under protocol number 78/2012–CEUA.

The lambs were born from Bergamasca ewes of 1 to 4 years of age that weighed 42 ± 1.0 kg during the proximate breeding season. Newborns remained with their mothers during the entire experimental period.

In the day of birth, all lambs were subjected to a physical examination and were weighted using scales (in kilograms), appeared to be normal and healthy. The information about animals body mass during the examination appears a more appropriate way to express such a relationship between bodyweight (BW) and electrocardiographic parameters. The body surface area (BSA) was calculated using the Thomas’s formula (BSA =10 · 1 × BW) [60, 61].

The electrocardiographic tests of the 22 non-anaesthetized lambs were initiated within the first 24 h of birth and were repeated once a week until 35 days of age. The tests were performed once a week, that is, on days 0, 7, 14, 21, 28, and 35 after birth.

### Electrocardiography

The ECG pattern was monitored with standard bipolar leads using de Einthoven’s triangle in the frontal plane, with conscious lambs being placed in a right lateral recumbent position, with the fore and hind limbs placed at right angles to the long axis of the body. The electrodes were attached to the forelegs (in the anterolateral skin fold situated immediately above the olecranon process; a red electrode was placed on the right leg and a yellow electrode was placed on the left leg) and the hind legs (in the skin fold at knee level; a green electrode on the left leg and a black electrode on the right leg) [38].

To record the base-apex lead, positive electrodes were attached to the left thorax in the fifth intercostal space, either at the elbow or where the apex beat was most readily palpated. The negative electrode was attached to the skin at the right jugular furrow, two-thirds of the way from the ramus of the mandible to the thoracic inlet. The ground electrode was attached to a site that was remote from the heart (such as the withers, i.e. the ridge between the shoulder blades) [62].

For each lamb, the recording was performed using a 12-channel electrocardiograph (ECG module on the Computer Module ECG-PC TEB; Tecnologia Eletronica Brasileira, São Paulo, Brazil), which was directly connected to an automatic microcomputer. A 50 mm/s paper speed was used, with a calibration of 10 mm/mV (1 mv = 1 cm). All readings were obtained simultaneously to reduce the length of time that the lambs were restrained (max. 90 s). The position of animal during ECG was right lateral decubitus.

The following parameters were analyzed in all leads. The parameters of ECG was calculated – by computer program: P and T waves, QRS complex, and the PR, QT, and RR intervals, to measure amplitude (mv) and duration(s). The morphology, heart rate (HR), cardiac rhythms, and polarity of the T wave were assessed. The mean electrical axis was calculated by using leads I and III, based on the method described [38]^.^ QTc values were obtained from the following equation: QTcV = QT + 0.087(1- RR) [63]. To evaluate P-wave dispersion (PWD) and QTc-interval dispersion (QTcID) in lambs was calculated as the difference between the maximum and the minimum P-wave duration, as the difference between the maximum and the minimum QTc duration, respectively.

### Statistical analyses

Nonparametric statistical analyses (Friedman test and Mann–Whitney test) were performed. The Friedman test was used to analyze any possible effects of time on each sex for each of the evaluated parameters. The Mann–Whitney test was used for the analysis of any possible effects at each time, with the level of significance was set at 5 %. Spearman’s test was used to visualize the degree of correlation between PR, QT, RR, and HR. The analysis was conducted using SAS 9.2. In addition, Pearson’s correlation was calculated between dependent variables among each time point assessed.

## References

[1] Amer-Wahlin I, Ingemarsson I, Marsal K, Herbst A (2005). Fetal heart rate patterns and ECG ST segment changes preceding metabolic acidaemia at birth. Inter J Obst Gynaecol.

[2] Brazilian Society of Cardiology. Diretrizes de Interpretação do eletrocardiograma de Repouso. Arq Bras Cardiol. 2003;80(Suppl3):1–18.

[3] Davignon A, Rautaharju P, Barselle E (1979). Normal ECG standards for infants and children. Pediatr Cardiol.

[4] Abreu DC, Feldman J, Deccache W, Goldwasser GP (2004). Elementos significativos do eletrocardiograma nos recém-natos e na primeira infância: suas utilidades clínicas. Revista da SOCERJ.

[5] Yiallourou SR, Sands SA, Walker AM, Horne R (2012). Maturation of heart rate and blood pressure variability during sleep in term-born infants. Sleep.

[6] Yiallourou SR, Witcombe NB, Sands SA, Walker AM, Horne SC (2013). The development of autonomic cardiovascular control is altered by preterm birth. Early Hum Dev.

[7] Pladys P, Arsenault J, Reix P, Lafond JR, Moreau-Bussière F, Praud JP (2008). Influence of prematurity on postnatal maturation of heart rate and arterial pressure responses to hypoxia in lambs. Neonatol.

[8] Cook CD, Drinker PA, Jacobson HN, Levison H, Strang LB (1963). Control of pulmonary blood flow in the foetal and newly born lamb. J Physiol.

[9] Dawes GS, Mott JC, Widdicombe JG (1955). The patency of the ductus arteriosus in newborn lambs and its physiological consequences. J Physiol.

[10] Tovar P, Santisteban R, Porras A, Castejon FM (1985). Electrocardiographic analysis of auricular electric systole in the sheep. Rev Esp Fisiol.

[11] Schultz RA, Pretorius PJ, Terblanche M (1972). An electrocardiographic study of normal sheep using a modified technique. Onderstepoort J Vet Res.

[12] Schultz RA, Coetzer JA, Kellerman TS, Naudé TW (1982). Observation on clinical, cardiac and histopathology effects of fluoroacetate in sheep. Onderstepoort J Vet Res.

[13] Zugaib M, Forsythe AB, Nuwayhid B, Lieb SM, Tabsh K, Erkkola R (1980). Mechanisms of beat-to-beat variability in the heart rate of the neonatal lamb. Influence of the autonomic nervous system. Am J Obstet Gynecol.

[14] Patural H, St-Hilaire M, Pichot V, Beuchée A, Samson N, Piccione G (2007). Physiological parameters in lamb during the first 30 days postpartum. Small Rum Res.

[15] Beuchée A, Hernández AI, Duvareille C, Daniel D, Samson N, Pladys P (2012). Influence of hypoxia and hypercapnia on sleep state dependent heart rate variability behavior in newborn lambs. Sleep.

[16] Sill B, Roy N, Hammer PE, Triedman JK, Sigg DC, Kelly Mark F (2011). Development of an ovine model of pediatric complete heart block. J Surg Res.

[17] Liebman J (2010). The normal electrocardiogram in the newborn and neonatal period and its progression. J Electrocardiol.

[18] Resende JG, Zaconeta CAM, Ferreira ACP, Silva CAM, Rodrigues MP, Rebello CM (2006). Avaliação do pico de pressão, do volume corrente e da frequência respiratória durante ventilação de carneiros prematuros utilizando balão auto-inflável. J Pediatr.

[19] Heir CR, Hampton TG, Wang D, DiDonato CJ (2010). Development of electrocardiogram intervals during growth of FVB/N neonate mice. BMC Physiol.

[20] Greiveldinger L, Veissier I, Boissy A (2009). Behavioural and physiological responses of lambs to controllable vs. uncontrollable aversive events. Psychoneuroendocrinology.

[21] Montoya AJA, Ponce VJ (1986). Normal cardiac rhythms in goats. MedVet.

[22] Pourjafar M, Badiei K, Chalmeh AA, Sanati AR, Shahbazi A, Badkobeh M (2013). Age-related cardiac arrhythmias in clinically healthy iranian najdi goats. Bulg J Vet Med.

[23] Rezakhani A, Ejtehadi M (1980). Some electrocardiographic parameters of the fat tailed sheep. Zen Bl Vet Med.

[24] Mir SA, Nazki AR, Raina R (2000). Comparative electrocardiographic studies and differing effects of pentazocine on ECG, heart and respiratory rates in young sheep and goats. Small Rum Res.

[25] Torío R, Cano M, Montes A, Prieto F, Benedito JL (1997). Comparison of two methods for electrocardiographic analysis in Gallega sheep. Small Rum Res.

[26] Ker J, Webb EC, Ker JA, Bekker PA (2003). Cardiac Memory T wave Frequency as an Electrocardiographic Surrogate for Structural Myocardial Alteration in the Hearts of Dorper Sheep. J Vet Res.

[27] Piccione G, Borruso M, Fazio F, Giannetto C, Caola G (2007). Physiological parameters in lambs during the first 30 days postpartum. Small Rum Res.

[28] Lima CCV, Silva DFM, Costa JN, Costa Neto AO, Souza TS (2010). Parâmetros fisiológicos de cordeiros mestiços (1/2 e ¾ Dorper) do nascimento aos 90 dias de idade. Rev Bras Saúde Prod Anim.

[29] Grundy SA (2006). Clinically relevant physiology of the neonate. Vet Clin North Am Small An Pract.

[30] Ferrer MI (1985). The evolution of the electrocardiogram in the developing heart. J Insur Med.

[31] Selig FA, Tonolli ER, Silva EVCM, Godoy MF (2011). Variabilidade da frequência cardíaca em neonatos prematuros e de termo. Arq Bras Cardiol.

[32] Cunningham JG (2008). Tratado de Fisiologia Veterinária.

[33] Duvareille C, Praud JP (2010). Postnatal autonomic activity in the preterm lamb. Res Vet Sci.

[34] Tanaka K, Zlochiver S, Vikstrom KL, Yamazaki M, Moreno J, Klos M (2007). Spatial distribution of fibrosis governs fibrillation wave dynamics in the posterior left atrium during heart failure. Circ Res.

[35] Berenfeld O, Ennis S, Hwang E, Hooven B, Grzeda K, Mironov S (2011). Time- and frequency-domain analyses of atrial fibrillation activation rate: The optical mapping reference. Heart Rhythm.

[36] Butters TD, Aslanidi OV, Zhao J, Smaill B, Zhang H (2013). A novel computational sheep atria model for the study of atrial fibrillation. Interface Focus.

[37] Zhao J, Krueger MW, Seemann G, Meng S, Zhang H, Dössel O (2012). Myofiber orientation and electrical activation in human and sheep atrial models. Conf Proc IEEE Eng Med Biol Soc.

[38] Tilley LP (1992). Essentials of canine and feline electrocardiography interpretation and treatment.

[39] Ferasin L, Amodo A, Murray JL (2010). Lack of correlation between canine heart rate and body size in veterinary clinical practice. J Small Anim Pract.

[40] Noujaim SF, Lucca E, Munoz V, Persaud D, Berenfeld O, Meijler FL (2004). From mouse to whale: a universal scaling relation for the PR interval of the electrocardiogram of mammals. Circulation.

[41] Milnor W (1979). Aortic wavelength as a eterminant of the relation between heart rate and body size in mammals. Ame J Physiol.

[42] Tovar P, Castejon FM, Santisteban R (1986). Analísis de la sístole eléctrica ventricular de ovinos. I Fenómeno de despolarización. J Am Vet Med Assoc.

[43] Tovar P, Castejon FM, Santisteban R (1986). Analísis de la sístole eléctrica ventricular de ovinos. II Repolarización. J Am Vet Med Assoc.

[44] Tovar P, Santisteban R (1987). Effects of maturational changes upon the orientation of auricular vector in sheep. J Am Vet Med Assoc.

[45] Tovar P, Santisteban R (1987). Maturation effects on the activation and recuperation ventricular vectors from sheep. J Am Vet Med Assoc.

[46] Keunen H, van Wijngaarden WJ, Sahota DS, Hasaart TH (2000). The PR interval-fetal heart rate relationship during repetitive umbilical cord occlusions in immature fetal sheep. Eur J Obstet Gynecol Reprod Biol.

[47] Atmaca N, Simsek O, Emre B (2014). Some electrocardiographic values of Angora goats. Ankara Oniv Vet Fak Derg.

[48] Westgate JA, Wassink G, Bennet L, Gunn AJ (2005). Spontaneous hypoxia in multiple pregnancies is associated with early fetal decompensation and enhanced T-wave elevation during brief repeated cord occlusion in near-term fetal sheep. Am J Obst Gynecol.

[49] El-Gamal A, Gallagher D, Nawras A, Gandhi P, Gomez J, Allison DV (1995). Effects of obesity on QT, RR and Qtc intervals. Am J Cardiol.

[50] Aytemir K, Ozer N, Atalar E, Sade E, Aksöyek S, Ovünç K (2000). P-wave dispersion on 12-lead electrocardiography in patients with paroxysmal atrial fibrillation. Pacing Clin Electrophysiol.

[51] Dilaveris PE, Gialafos EJ, Sideris SK, Theopistou AM, Andrikopoulos GK, Kyriakidis M (1998). Simple electrocardiographic markers for the prediction of paroxysmal idiopathic atrial fibrillation. Am Heart J.

[52] Bu’Lock FA, Mott MG, Martin RP (1995). Left ventricular diastolic function in children measured by Doppler echocardiography: normal values and relation with growth. Br Heart J.

[53] de Simone G, Daniels SR, Devereux RB, Meyer RA, Roman MJ, de Divitiis O (1992). Left ventricular mass and body size in normotensive children and adults: assessment of allometric relations and impact of overweight. J Am Coll Cardiol.

[54] Zhendong Y (1998). Effects of age and respiration on right ventricular diastolic filling patterns in normal children. Pediatr Cardiol.

[55] Maron BJ, Leyhe MJ, Casey SA, Gohman TE, Lawler CM, Crow RS (2001). Assessment of QT dispersion as a prognostic marker for sudden death in a regional nonreferred hypertrophic cardiomyopathy cohort. Am J Cardiol.

[56] Zaidi M, Robert A, Fesler R, Derwael C, Brohet C (1996). Dispersion of ventricular repolarization in hypertrophic cardiomyopathy. J Electrocardiol.

[57] Vialle E, Albalkhi R, Zimmerman M, Friedli B (1999). Normal values of signal-averaged electrocardiographic parameters and QT dispersion in infants and children. Cardiol Young.

[58] Akyuz A, Alpsoy S, Akkoyun DC, Nalbantoglu B, Ozdilek B, Donma MM (2013). Does low birth weight affect P-wave and QT dispersion in childhood?. Pacing Clin Electrophysiol.

[59] Nigro G, Russo V, Rago A, Papa AA, Cioppa ND, Di Meo F (2012). Heterogeneity of ventricular repolarization in newborns with severe aortic coarctation. Pediatr Cardiol.

[60] Price GS, Frazier DL (1998). Use of Body Surface Area (BSA)-Based Dosages to Calculate Chemotherapeutic Drug Dose in Dogs: I. Potential Problems with Current BSA Formulae. J Vet Intern Med.

[61] Hill RC, Scott KC (2004). Energy requirements and body surface area of cats and dogs. J Am Vet Med Assoc.

[62] Kojouri GA, Rezakhani A, Ahmadi H (2009). Arrhythmias in advance stiff lamb disease. Small Rum Res.

[63] Van de Water A, Verheyeu J, Xhoneux R, Reneman R (1989). An improved method to correct the QT interval of the electrocardiogram for changes in heart rate. J Pharmacol Methods.

